# Care of Children with DiGeorge Before and After Cultured Thymus Tissue Implantation

**DOI:** 10.1007/s10875-021-01044-0

**Published:** 2021-05-18

**Authors:** Stephanie E. Gupton, Elizabeth A. McCarthy, M. Louise Markert

**Affiliations:** 1 Department of Pediatrics, Division of Allergy and Immunology, Duke University Medical Center, Durham, NC USA; 2 Department of Immunology, Duke University Medical Center, Durham, NC USA

**Keywords:** Congenital athymia, Complete DiGeorge anomaly, CHARGE syndrome, 22q11.2 deletion syndrome, Cultured thymus tissue implantation

## Abstract

**Background:**

Children with complete DiGeorge anomaly (cDGA) have congenital athymia plus a myriad of other challenging clinical conditions. The term cDGA encompasses children with congenital athymia secondary to 22q11.2DS, CHARGE syndrome (coloboma, heart defects, choanal atresia, growth or mental retardation, genital abnormalities, and ear abnormalities and/or deafness), and other genetic abnormalities. Some children have no known genetic defects. Since 1993, more than 100 children with congenital athymia have been treated with cultured thymus tissue implantation (CTTI). Naïve T cells develop approximately 6 to 12 months after CTTI. Most of the children had significant comorbidities such as heart disease, hypoparathyroidism, and infections requiring complex clinical care post cultured thymus tissue implantation (CTTI).

**Objective:**

The purpose of this guidance is to assist multidisciplinary teams in caring for children with cDGA both before and after CTTI.

**Methods:**

Thirty-one specialists, in addition to the authors, were asked to share their experience in caring for children with cDGA at Duke University Health System, before and after CTTI. These specialists included physicians, nurses, dentists, therapists, and dieticians.

**Results:**

The goal of a multidisciplinary approach is to have children in the best possible condition for receiving CTTI and provide optimal care post CTTI through development of naïve T cells and beyond. The CTT (cultured thymus tissue) must be protected from high doses of steroids which can damage CTT. Organs must be protected from adverse effects of immunosuppression.

**Conclusion:**

Creating a multidisciplinary team and a detailed plan of care for children with cDGA is important for optimal outcomes.

## Introduction

Cultured thymus tissue implantation (CTTI) is an investigational procedure that has been used at Duke University Health System since 1993 to treat more than 100 children with congenital athymia, most of whom had complete DiGeorge anomaly (cDGA) [1–3]. These children presented with a myriad of medical issues, the most common of which were heart defects and hypoparathyroidism [2]. The purpose of this guidance is to provide best practices for management of these children both before and after CTTI.

## Methods

All children received CTTI under Institutional Review Board–approved protocols after parental consent. Because these children have many clinical issues, this guidance is presented based on organ systems. The immunology section includes both pre- and post-CTTI-specific care as immunologic outcomes are directly impacted by CTTI. Thirty-one clinical specialists were consulted for contributions to this guidance document.

## Results

### Immunology

Diagnosis: In the USA, newborn screening of T cell receptor rearrangement excision circles (TRECs) identifies children with congenital athymia [4, 5]. Upon receiving the report of low TRECs on a newborn screen, an immunologist should be consulted, protective isolation initiated, and breast-feeding stopped until the diagnosis of athymia is ruled out. No vaccines should be given. If needed, use only leukoreduced, CMV-negative, irradiated blood products. For the diagnosis of cDGA, a lymphocyte enumeration must show very low (< 50/mm^3^) naïve T cells with B and NK cells present. Patients with severe combined immunodeficiency (SCID) can be identified by a SCID genetic panel or whole exome sequencing (WES). Complete DiGeorge anomaly can also be distinguished from SCID using thymic organoid cultures, currently available in two research laboratories [6, 7]. The criteria for diagnosing congenital athymia are in Table 1.

**Table 1:** Criteria for the diagnosis of athymia

Criteria required for the diagnosis of athymia • Total CD3 T cells < 50/mm^3^ OR naïve T cells < 50/mm^3^ or naïve T cells < 5% of total T cells • Presence of B and NK cells • Absence of SCID genetic defects based on SCID genetic panel or whole exome sequencing ▪ Thymic organoid cultures [6, 7] may be used if diagnosis is unclear due to phenotype Antibodies used to detect naïve T cells • CD45RA x CD45RO • CD45RA x CD62L • CD45RA x CD31	

Pre-CTTI management: Children identified with congenital athymia should receive immunoglobulin replacement to maintain immunoglobulin G (IgG) trough levels of at least 800 mg/dl [8–10]. Subcutaneous immunoglobulin is often preferred over intravenous immunoglobulin (IVIG) to avoid complications associated with central lines. Prophylactic medications should be started to prevent infections (see infectious disease section). Lymphocyte enumerations should be obtained every 6 months prior to CTTI.

Hospitalized children with congenital athymia should remain in protective isolation. Once discharged, minimize exposure to people other than immediate family by using excellent hand hygiene, limiting visitors, homeschooling other young children, and family members showering/changing clothes upon reentry to the home from work.

Autologous graft versus host disease (aGVHD) with immune dysregulation: From birth to 6 to 12 months post CTTI, children with congenital athymia may develop aGVHD from uneducated recipient T cells that did not develop in a thymus [2]. The clinical presentation of aGVHD varies but usually involves the skin and gut. Skin rashes are often accompanied by lymphadenopathy [11]. Skin biopsy is necessary to confirm the diagnosis of aGVHD (see the “Dermatology’ section). For persistent diarrhea, consider gut biopsies. Total parenteral nutrition (TPN) may be required.

For suspected aGVHD, a complete blood count, differential, and lymphocyte enumeration are indicated. T cell counts can increase to over 27,000/mm^3^. These T cells do not express naïve T cell or recent thymus emigrant (RTE) markers [11].

The liver and lungs are infrequent targets of uneducated T cells of aGVHD. However, if serum aminotransferases increase to over 150 U/L, liver biopsy should be considered to confirm the diagnosis before starting immunosuppression [12]. If lungs are involved, infection should be ruled out before starting immunosuppression.

The first-line treatments of the rash in children with aGVHD are topical moisturizers, steroid creams, and/or calcineurin inhibitor creams. If topical treatments are ineffective, low doses of a systemic calcineurin inhibitor (cyclosporine, tacrolimus) may be needed.

In severe cases, cyclosporine trough levels of 180 to 220 ng/ml or tacrolimus trough levels of 7 to 10 ng/ml may be required. In rare cases, such as recurrence of previously controlled aGVHD, trough levels may be increased to 250 to 300 ng/ml for cyclosporine and 10 to 15 ng/ml for tacrolimus.

If calcineurin inhibitors are not tolerated, e.g., resulting in hypertension and/or low glomerular filtration rate (GFR), steroids can be used at the lowest effective dose. In severe cases, 1 to 2 mg/kg/day of methylprednisolone may be needed to decrease symptoms. Pulse steroids (methylprednisolone of 30 to 40 mg/kg/day for 3 days) should never be given post CTTI as the pulse can permanently damage the CTT [13].

CTTI: CTTI procedures have been reported in multiple publications [14, 15]. Rice et al. (2004) describe the surgical procedure [14]. Briefly, an incision is made in the skin and the surface of the quadriceps is exposed. CTT is placed in “pockets” made in the quadriceps muscle, chosen for its good blood supply to provide oxygen and nutrients to CTT.

Post CTTI: Naïve T cells usually develop 6 to 12 months post CTTI [1]. Thus, upon return to the referring center, clinical care is essentially identical to that prior to CTTI.

Children can develop aGVHD at any time prior to naïve T cell development. They should be evaluated weekly for the first 2 months and at least monthly for the remainder of the first year. Lymphocyte enumerations should be done every 3 months until naïve T cells are greater than 10% of the total T cells and at 12 months post CTTI. When naïve T cells are over 10% of total T cells, the calcineurin inhibitor is weaned over approximately 10 weeks. Prophylactic medications, immunoglobulin replacement, and protective isolation can usually be stopped by 1 year if all criteria in Table 2 are met.

**Table 2:** Guidelines for stopping *Pneumocystis jiroveci* pneumonia (PJP) prophylaxis and immunoglobulin replacement

PJP, fungal, and mycobacterial prophylaxis will be discontinued when the CTTI recipient: • Is at least 9 months post transplantation • Phytohemagglutinin (PHA) response is > 100,000 counts per minute (cpm) • CD4^+^ count is > 200/mm^3^ • Off immunosuppression Immunoglobulin replacement will be discontinued initially when the CTTI recipient meets the following criteria: • Is at least 9 months post transplantation • PHA response is > 100,000 cpm • Off immunosuppression Permanent discontinuation of immunoglobulin replacement is recommended, if the trough IgG level, 2 months from last immunoglobulin dose, is within or above the normal range for age Restart immunoglobulin replacement if the trough IgG level is below the normal range for age at 2 months after last immunoglobulin dose • If immunoglobulin replacement is restarted, the approach is repeated 6 months to a year later with testing as above	

After CTTI, children with congenital athymia develop improved T cell numbers although usually less than the 10th percentile for age [1]. The children do well and are immunologically similar to the 10% of the general population and children with partial DGA (pDGA) who have counts below the 10th percentile for age.

After the first year post CTTI, lifetime annual evaluations should include assessment of T cell counts and monitoring for long-term adverse events including autoimmune disease, osteopenia, renal damage, and scoliosis. Recommended annual tests post CTTI are in Table 3.

**Table 3:** Annual evaluations beginning 2 years after CTTI

• Physical examination including height and weight • Complete blood count with differential • Lymphocyte enumeration • Serum immunoglobulins (IgG, IgA, IgM, and IgE) • Chemistry including sodium, potassium, glucose, BUN, and creatinine • Urinalysis • Urine calcium and urine creatinine • Liver function studies • Albumin • Calcium • Thyroid testing: (thyroid stimulating hormone and free thyroxine)	

Live vaccines were not given early in the CTTI program but more recently have been given once patients are off immunosuppression and demonstrate the ability to mount an immune response to inactivated vaccines. Live vaccines have been well tolerated [3]. Check titers to vaccines to insure that the patient is protected, especially to varicella and measles. Guidances for administration and timing of immunizations are in Tables 4 and 5.

**Table 4:** Immunization criteria

• Inactivated (killed) immunizations may be given when: ° Cellular proliferation to phytohemagglutinin > 100,000 cpm ° CD4^+^ count > 200/mm^3^ ° Naïve T cells > 10% with child off immunosuppression ° Trough IgG level within or above the normal range after a 2 month trial off immunoglobulin replacement • The reasons to not give immunizations prior to meeting the above criteria are that immunizations: ° Are not effective prior to development of immune function ° Can lead to disease if a live vaccine is inadvertently given prior to meeting the above criteria, and ° Can trigger cytopenias prior to and in the first year after implantation

**Table 5:** Guide for immunization administration after CTTI and after meeting the criteria in Table 4

• Start with inactivated (killed) vaccines • No more than 2 vaccines per month • Live vaccines are given after all the inactivated (killed) vaccines have been given and if CD4 > CD8 counts and CD8 > 100/mm3 ° Start with the varicella vaccine. If lesions develop that are problematic (which is rare), acyclovir may need to be given. ° Give the measles/mumps/rubella (MMR) vaccine 2 months after the varicella vaccine if there is no lingering reaction to the varicella vaccine. ° After the MMR do not give other live vaccines for 6 months as 1 month post the MMR, the T cell count may drop by 50% [16].	

### Cardiology

A baseline echocardiogram is recommended when there are signs of cardiac involvement [2]. Blood pressures should be maintained below the 90th percentile for age. Cardiac surgery, if indicated, should not be delayed while awaiting CTTI. Of note, wound healing is normal in children with congenital athymia/cDGA. Generally, CTTI is not performed in children with unrepaired cyanotic congenital heart disease due to the risk of hemodynamic instability and need for surgical intervention requiring high dose steroids in the immediate pre-/post-CTTI period. High-dose steroids are contraindicated in the immediate post CTTI period as they can damage the CTT. CTTI should not be performed in children with anticipated cardiac intervention within 2 months of CTTI.

### Dentistry

Excellent oral hygiene is needed to prevent dental or gingival problems. In children with concern for cavities, a dental consult should be obtained prior to CTTI. Confirm with the cardiologist if bacterial endocarditis prophylaxis is indicated. The dentist will provide guidance about side effects of medications, such as cyclosporine, that can cause gingival hyperplasia.

### Dermatology

A new rash in a child with congenital athymia/cDGA can be indicative of aGVHD. A skin biopsy, with T cells present, is necessary to confirm the diagnosis [11]. If topical steroids were applied prior to the biopsy, this can alter the histologic findings. If no T cells are present in the initial biopsy, allow the rash to evolve and repeat the biopsy. Classically there is exocytosis, parakeratosis and spongiosis identified by pathology. Dyskeratosis and satellite cell necrosis can be seen [17]. It is important to start immunosuppression for severe rashes associated with aGVHD. Topical steroids or calcineurin inhibitors can be used as a first-line treatment. In many cases, a systemic calcineurin inhibitor is required to control the rash.

### Endocrinology

Many children with cDGA have persistent hypoparathyroidism [1, 2]. Hypoparathyroidism can be present at birth but often develops around 5 weeks after birth. Unlike many children with pDGA, hypoparathyroidism in children with cDGA is usually a lifelong problem [18–23]. With stressors such as acute illness, infections, or surgery, calcium requirements can increase dramatically. Children are usually treated with calcitriol and calcium (see Table 6). Some centers may use synthetic parathyroid hormone [24].

**Table 6:** Treatment of hypoparathyroidism

• Give calcitriol (1.25 di OH vitamin D) with daily dosing of 30 to 80 ng/kg. Ideally, calcitriol should be given 30 to 60 min prior to the calcium especially in children needing high doses. • Give calcium with an elemental dose of up to 50 mg/kg/day. ° Calcium carbonate (40% elemental) is not absorbed if the patient is on a proton pump inhibitor. ° Other options are calcium acetate (25.3% elemental), calcium citrate (21.1% elemental), or calcium chloride (27.2% elemental). • Monitor urine calcium to creatinine ratio ° Urine calcium to creatinine ratio is followed to prevent nephrocalcinosis. ▪ The ratio should be < 0.2 if over 12 months and < 0.5 for children under 12 months. ▪ Decrease calcium as necessary to prevent nephrocalcinosis. ▪ Increase calcium as necessary to prevent osteopenia/fractures. ▪ Note: the urine Ca/Cr ratio is altered by diuretics. • The target ionized calcium is usually 1.0 to 1.15 mmol/L with normal magnesium and phosphorus. The target ionized calcium can be higher if the urine calcium to creatinine ratio remains in the appropriate range (see above). ° Frequency of testing the urine to avoid nephrocalcinosis or osteopenia/fractures: ▪ Weekly to monthly in young children ▪ Every 3 months in older children	

Thyroid disease is present in approximately 30% of children with cDGA and can occur before or after CTTI [2]. Arrest of linear growth can be an early sign of hypothyroidism; therefore, length should be followed regularly. Hyperthyroidism is less frequent. A thyroid panel is recommended prior to CTTI and then every 6 months through 12 months post CTTI and then annually to assess for thyroid disease.

### Gastroenterology/Hepatology/Pancreas

Children with cDGA may present with suboptimal growth and nutritional status related to feeding difficulties such as dysphagia, enteropathy, gastrointestinal dysmotility (constipation), and/or other medical comorbidities [18, 21, 23, 25–34]. The majority of children have involvement of the gastrointestinal tract and require enteral nutritional support via tube feeding [35, 36]. It is important to avoid obesity from overfeeding and maintain a healthy body mass (weight for length).

Dysphagia with gagging/regurgitation of nipple feeds and risk of aspiration has been described on videofluoroscopic swallow studies in affected children independent of palatal anatomy [25, 26, 37]. Speech-language pathologists should evaluate efficiency and safety of swallow when indicated.

Diarrhea may be associated with enteropathy, infection, or may be a secondary effect of medications/antibiotics. Biopsies of the upper and lower GI tract should be considered to assess diarrhea and also hypoalbuminemia that is present without a cause. Mucosal apoptosis can be seen with aGVHD. Constipation is common requiring appropriate laxative regimens.

Elevated serum aminotransferase levels may indicate T cell–mediated hepatitis, and levels should be performed monthly for 3 months and then every 3 months through 12 months post CTTI. When other etiologies for elevated enzymes are not found (hepatotoxic medications or infection), liver biopsy may be necessary to identify T cells in the parenchyma [12] or evidence of autoimmune hepatitis prior to starting immunosuppression.

For children on calcineurin inhibitors who develop new gastrointestinal symptoms (especially abdominal pain), pancreatitis must be considered. Serum lipase should be monitored.

### Genetics

It is important to rule out SCID using a genetic SCID panel or whole-exome sequencing as coexistent SCID and DiGeorge anomaly have been reported. Multiple genes are implicated in congenital athymia as found in cDGA or Foxn1 deficiency (see Table 7).

**Table 7:** Genetic findings in congenital athymia

Genetic defect	Test
22q11.2 deletion syndrome [27, 38–41]	Chromosome microarray, fluorescence in situ hybridization (FISH) for deletions
Recurrent microdeletions at chromosome 2p11.2 [42]	Chromosome microarray, fluorescence in situ hybridization (FISH) for deletions
Chromodomain helicase DNA binding protein 7 (CHD7), CHARGE syndrome (coloboma, heart defects, choanal atresia, growth or mental retardation, genital abnormalities, and ear abnormalities and/or deafness) [28, 33, 43–45]	Whole-exome sequencing or sequencing of CHD7
Forkhead box N1 (Foxn1) [46–48]	Whole-exome sequencing
T-box transcription factor 1 (TBX-1) [49, 50]	Whole-exome sequencing
T-box transcription factor 2 (TBX-2) [51]	Whole-exome sequencing
Paired box 1 (PAX1) [52]	Whole-exome sequencing
Semaphorin 3 (SEMA3E) [53]	Whole-exome sequencing
10p deletions [54]	Chromosome microarray, fluorescence in situ hybridization (FISH) for deletions

### Growth and Development

Children with cDGA, as those with pDGA, usually have developmental delay though the severity can vary widely [18, 22, 28, 30, 31]. Early intervention with physical, occupational, and speech therapies is critical to optimizing development. Scoliosis can start at a very young age in these children, so monitoring is essential to identify the curve early so that bracing can be initiated [55]. Behavioral and psychiatric problems are common [22, 23, 26, 55–61]. Therapies should not be delayed while waiting for CTTI.

### Hematology

Cytopenias (thrombocytopenia and anemia) due to immune dysregulation are common before and in the first year after CTTI as in pDGA patients [62, 63]. Neutropenia is less frequent. Cytopenias may also be due to marrow suppression from intercurrent infections or medications.

Some children develop coagulopathies related to infections, nutritional deficiencies, or liver dysfunction. They may require central venous access and prolonged hospitalizations which increase risk of thrombosis.

### Infectious Disease

Prophylactic antibiotics and antifungals should be started as described below. As soon as the diagnosis of congenital athymia is made, fluconazole (3 to 6 mg/kg/day) and azithromycin (20 mg/kg weekly) should be started. Azithromycin given weekly can cause significant increases in calcineurin inhibitor levels leading to kidney and liver damage. Once daily dosing (5 mg/kg/dose) can be considered in patients on calcineurin inhibitors. After 1 month of life, trimethoprim-sulfamethoxazole should be started at prophylaxis dosing. Family members should be up to date on vaccinations including seasonal vaccines, as age appropriate, with the exception of rotavirus and varicella vaccines, which should not be administered in siblings of immunocompromised patients.

Mycobacterial infections (*Mycobacterium avium* complex and *M. kansasii*) have been identified in a small number of children prior to development of naïve T cells. Mycobacterial infections require multiple anti-microbials and a prolonged treatment course for resolution. Children with mycobacterial infection should not be treated with steroids prior to the development of naïve T cells.

Vaccines should not be given prior to development of naïve T cells. See Tables 4 and 5 for guidelines. See Table 8 for treatment after exposure to varicella or measles.

**Table 8:** Plan for post-exposure prophylaxis to varicella or measles prior to vaccine administration

Exposure	Post-exposure prophylaxis
Varicella	If a child is exposed to varicella, the child should be given intramuscular varicella zoster immunoglobulin or intravenous immunoglobulin within 3 days of exposure. If the child develops chickenpox spots (after the 10 to 21 day incubation period), the child should be admitted to the hospital and receive intravenous acyclovir
Measles	IVIG can be given after exposure to measles but its efficacy is not proven

### Invasive Procedures

Careful preoperative preparation is essential and should involve surgeons, anesthesiologists and radiologists as indicated. Complete review of medical conditions, medications, electrolyte disturbances, cardiac function, and pulmonary status is critical. A comprehensive anesthesia plan should be made prior to any procedure, especially CTTI, to ensure the anesthesiologist, surgeon, and medical staff are aware of the underlying medical issues (in particular congenital heart disease and airway anomalies) and medications are administered or discontinued as appropriate prior to the procedure. The anesthesiologist should be aware that steroids should be avoided, if possible, because of the potential harm to the CTT.

It is important to keep the calcium stable during surgeries. An ionized calcium should be obtained 4 h prior to surgery to ensure adequate calcium levels. Even in children who normally require ionized calcium levels < 1.0 mmol/L to prevent nephrocalcinosis, a higher level of 1.5 to 2 mmol/L is desirable during operative procedures.

Peripherally inserted central catheters (PICC) are preferred for children needing short-term central venous access around the time of implantation. For children requiring central lines for clinical care such as TPN, frequent blood draws or longer term intravenous immunosuppression, the type of access should be based on the needs and characteristics of the patient (i.e., age, weight, length of time line is needed, potential for infection). Wound healing can be suboptimal in children who are on steroids; special attention should be given to securing external lines. The central line should be looped under a secure dressing to decrease the risk of the line being dislodged and may be tunneled out the back in an active child.

### Nephrology/Urology

Congenital kidney and urinary tract anomalies exist in 30% of children with cDGA as in pDGA [64, 65]. These anomalies include renal hypoplasia or dysplasia, obstruction, reflux, and unilateral renal agenesis. Children with cDGA with renal hypoplasia, dysplasia, or unilateral renal agenesis are especially vulnerable to renal insufficiency if on calcineurin inhibitors. For certain anomalies, surgical procedures such as re-implantation of ureters or repair of fistulas between the rectum and the bladder may be necessary [66]. Timing of these repairs should be per urologic surgical standards.

Nephrology consultation is often indicated. The glomerular filtration rate (GFR) should be assessed with an initial nuclear medicine study. Renal monitoring should include BUN, creatinine, cystatin-C, and proteinuria (urine protein to creatinine ratio). Proteinuria is a marker of renal disease. Children with underlying renal hypoplasia/dysplasia can have proteinuria prior to CTTI. After CTTI, common causes for proteinuria are calcineurin inhibitors and other renal toxic medications such as vancomycin, aminoglycosides, and cidofovir. Avoid nephrotoxic medications and iodinated contrast when possible. Dosing and/or medication adjustments may be required to protect the kidneys, for instance, using linezolid instead of vancomycin. Occasionally, proteinuria associated with nephrotic syndrome can be caused by auto-immune-associated glomerular disease and may require a kidney biopsy [67]. Serum creatinine and urinalysis should be performed monthly for 3 months and then every 3 months through 12 months post CTTI. Lastly, nephrocalcinosis can cause more renal damage and may occur due to medical therapies for hypocalcemia. Monitoring for hypercalciuria and, when appropriate, obtaining a renal ultrasound to evaluate/monitor nephrocalcinosis may be indicated.

### Neurology/Psychiatry

Neurologists and psychiatrists are important for care of children with cDGA. Brain magnetic resonance imaging may be useful in evaluating children with CHARGE and 22q11.2DS for significant abnormalities [68]. Occasionally children with cDGA have seizures. Neurologists can help determine the etiology for the seizures and manage the seizure disorder. Seizures can also be related to hypocalcemia especially in infancy. Psychiatric disease such as schizophrenia and bipolar disorder can develop in the teen years or later [69].

### Ophthalmology

An ophthalmology examination should be done to rule out coloboma in children with CHARGE [31–33]. Microphthalmos is common in CHARGE syndrome [26, 28, 30]. Hooded eyelids and hypertelorism are seen in children with 22q11.2DS [61].

### Otolaryngology (ENT)

Children with cDGA can have multiple problems involving the head and neck. A hearing evaluation should be obtained early (often under anesthesia) in children with CHARGE. Hearing aids should be ordered as soon as possible to facilitate speech and language development. Children with middle ear effusions may benefit from myringotomy tubes. For severe sensorineural hearing loss, cochlear implants can be considered, preferably once T cells develop post CTTI.

Children with CHARGE are often born with choanal atresia, a challenging condition that frequently recurs after the initial dilation surgery necessitating additional dilation procedures [26, 30, 32, 33].

Children may have recurrent or chronic sinus infections due to immune deficiencies. These may be refractory to standard medical management and require surgical intervention. In those instances, a computed tomography (CT) scan of the sinuses can help assess the degree and severity of the underlying sinus condition.

A CT assessment of the semicircular canals is important for children with CHARGE [30]. This allows the physicians to provide anticipatory guidance related to problems with balance in the child as they reach the age to sit, stand and walk.

Children with structural airway abnormalities may have obstructive sleep apnea. Adenoidectomies and tonsillectomies are sometimes recommended for management. It is important to note that there can be medial displacement of the carotid artery in children with cDGA. This can increase the risk of life-threatening bleeding with tonsillectomy or adenoidectomy; therefore, when the anatomy is in question, computerized tomography angiography should be performed to determine the location of the carotid arteries prior to surgery [70]. Complete DGA associated with certain genetic defects can include velopharyngeal insufficiency which can be worsened by adenoidectomy [23].

Children with 22q11.2DS and CHARGE often present with cleft lip and/or palate [18, 22, 23, 26, 28, 30–33]. The cleft lip and cleft palate repair may be delayed until after CTTI. Children with velopharyngeal insufficiency without overt cleft palate (common among 22q11.2DS) require a multidisciplinary workup including speech pathology, plastic surgery, and otolaryngology often in context of a cleft team. Many will require surgical intervention in addition to speech therapy.

### Pulmonary

Children with cDGA can have bronchomalacia, atelectasis, recurrent pneumonia and occasionally pulmonary fibrosis [71–73]. If a child develops increased work of breathing, hypoxia, or a chronic cough, a chest radiograph should be done to rule out pneumonia or atelectasis. A chest CT scan is often necessary if the etiology is not apparent on a chest radiograph. Persistent atelectasis or recurrent pneumonia may require bronchoscopy to diagnose bronchomalacia. A broncho-alveolar lavage can be performed during the bronchoscopy to identify infectious organisms. If the chest CT shows evidence of interstitial lung disease, a lung biopsy may be necessary.

For children requiring long-term mechanical ventilation, tracheostomy should be considered as it allows for a stable airway without sedation and may allow for successful ventilator weaning. Without sedation, these children can have better developmental outcomes.

### Psychosocial/Family

Caring for a child with disabilities can be very stressful. The parents/family may need psychosocial support and mental health services. Referrals to mental health providers that utilize resilience-based interventions provide opportunities for strategies to improve coping and family functioning. Psychosocial support from child life specialists can help reduce the stress associated with hospitalization.

Having a family member with a chronic illness can have a significant negative impact on the entire family unit [74]. Involving siblings in the care of the child can help affirm that they are important members of the family.

## Discussion

Children with congenital athymia should have a medical home, preferably in a health system with specialists knowledgeable in the underlying problems of the children both before and after CTTI. Physicians, nurses, and therapists need to be aware of the multiple medical issues to provide comprehensive care for these children. Currently there are many 22q11.2DS and CHARGE clinics in the USA. After CTTI and development of naïve T cells, these clinics are a good resource for the families.

After the children develop normal T cells and complete their immunizations, families may require reminders about annual evaluations. These evaluations are important to detect new issues allowing referral to the appropriate specialists. Children with cDGA have developed hypocalcemia, arthritis, thyroid disease, and psychiatric disease later in childhood similar to patients with pDGA who also can develop these conditions later in life [55].

The immunologist should continue to follow the patient for life. Although the first patient who received CTTI is now over 20 years old, it is important to realize that the stability of T cell counts after this time period is not yet known.

## Data Availability

Not applicable.

## Abbreviations

aGVHD: Autologous graft versus host disease
CHARGE syndrome: Coloboma, heart defects, choanal atresia, growth or mental retardation, genital abnormalities, and ear abnormalities and/or deafness
cDGA: Complete DiGeorge anomaly
CT: Computed tomography
cpm: Counts per minute
CTTI: Cultured thymus tissue implantation
CTT: Cultured thymus tissue
GFR: Glomerular filtration rate
IgG: Immunoglobulin G
IVIG: Intravenous immunoglobulin
MMR: Measles/mumps/rubella
PTH: Parathyroid hormone
pDGA: Partial DiGeorge anomaly
PHA: Phytohemagglutinin
PJP: *Pneumocystis jiroveci* pneumonia
SCID: Severe combined immunodeficiency
TRECs: T cell receptor rearrangement excision circles
TPN: Total parenteral nutrition
